# 

**Published:** 1916-03

**Authors:** 


### CONTENTS, MARCH, 1916—VOLUME XXXI, NO. 9.

### ORIGINAL ARTICLES.

Page

How Will the War Now Raging Be of Benefit to Mankind? By F. Paschal, M. D., San Antonio, Texas............ 351

Surgical Treatment of Retro-Displacements of the Uterus. By Dr. James A. Hill, Houston, Texas.............. 354

A Contribution to the Study of Hernias of the Ovary, of the Fallopian Tube and of the Ovary and Fallopian Tube. By Aime Paul Heineck, Chicago, Ill................ 356

Are Dents in the Forehead Inherited? By Dr. Leonard Keen Hirshberg, A. B., M. A., M. D., Johns Hopkins, Baltimore, Md.................................. 365

Contents for the April Issue........................... 366

### EDITORIAL DEPARTMENT.

An Important Announcement.......................... 367

What Organization May Accomplish..................... 368

Advance in Surgery..................................... 370

Medical Inspection .................................... 371

Cities Without Flies.................................... 371

Collections and Common Sense......................... 372

Items of General Interest............................... 374

Personals ............................................. 377

New Hospitals ......................................... 379

### ABSTRACTS AND SELECTIONS.

Diseases of the Epididymis and Testicle.................. 380

Fewer Doctors Turned Out............................. 382

The Campaign Against Cancer in Missouri................ 384

An Important Announcement........................... 385

Change of Location..................................... 388

Deaths ................................................ 388

Helpful Hints for the Busy Doctor...................... 390

